# *Trypanosoma cruzi* Modulates PIWI-Interacting RNA Expression in Primary Human Cardiac Myocytes during the Early Phase of Infection

**DOI:** 10.3390/ijms21249439

**Published:** 2020-12-11

**Authors:** Kayla J. Rayford, Ayorinde Cooley, Ashutosh Arun, Girish Rachakonda, Yulia Kleschenko, Fernando Villalta, Siddharth Pratap, Maria F. Lima, Pius N. Nde

**Affiliations:** 1 Department of Microbiology, Immunology and Physiology, Meharry Medical College, Nashville, TN 37208, USA; krayford@mmc.edu (K.J.R.); acooley@mmc.edu (A.C.); aarun@mmc.edu (A.A.); grachakonda@mmc.edu (G.R.); fvillalta@mmc.edu (F.V.); mlima@med.cuny.edu (M.F.L.); 2 Martsinovsky Institute of Medical Parasitology, Tropical and Vector Borne Diseases, Sechenov University, 119435 Moscow, Russia; ykleschenko@gmail.com; 3 School of Graduate Studies and Research, Bioinformatics Core, Meharry Medical College, Nashville, TN 37208, USA; spratap@mmc.edu; 4 Department of Molecular Cellular and Biomedical Sciences, School of Medicine, The City College of New York, New York, NY 10031, USA

**Keywords:** Chagas disease, human cardiac myocytes, fibrosis, parasite pathogenesis, piRNAs, piRNome, *Trypanosoma cruzi*

## Abstract

*Trypanosoma cruzi* dysregulates the gene expression profile of primary human cardiomyocytes (PHCM) during the early phase of infection through a mechanism which remains to be elucidated. The role that small non-coding RNAs (sncRNA) including PIWI-interacting RNA (piRNA) play in regulating gene expression during the early phase of infection is unknown. To understand how *T. cruzi* dysregulate gene expression in the heart, we challenged PHCM with *T. cruzi* trypomastigotes and analyzed sncRNA, especially piRNA, by RNA-sequencing. The parasite induced significant differential expression of host piRNAs, which can target and regulate the genes which are important during the early infection phase. An average of 21,595,866 (88.40%) of clean reads mapped to the human reference genome. The parasite induced 217 unique piRNAs that were significantly differentially expressed (q ≥ 0.8). Of these differentially expressed piRNAs, 6 were known and 211 were novel piRNAs. In silico analysis showed that some of the dysregulated known and novel piRNAs could target and potentially regulate the expression of genes including NFATC2, FOS and TGF-β1, reported to play important roles during *T. cruzi* infection. Further evaluation of the specific functions of the piRNAs in the regulation of gene expression during the early phase of infection will enhance our understanding of the molecular mechanism of *T. cruzi* pathogenesis. Our novel findings constitute the first report that *T. cruzi* can induce differential expression of piRNAs in PHCM, advancing our knowledge about the involvement of piRNAs in an infectious disease model, which can be exploited for biomarker and therapeutic development.

## 1. Introduction

The protozoan hemoflagellate parasite, *Trypanosoma cruzi*, is the causative agent of Chagas heart disease. Originally endemic in Mexico and South America, this disease is now present in most industrialized countries due to globalization and international travel [1,2,3,4,5]. Chagas disease causes severe morbidity and mortality worldwide. Therefore, it is now considered a new global health challenge [6]. In the US, the disease burden and risk of various forms of autochthonous transmission, in states sharing a border with Mexico and inland states, including in utero transmission, is higher than previously reported [4,7,8]. This renders Chagas disease an unfolding tragedy in the US [3]. Approximately 30% of *T. cruzi* infected individuals eventually present with incurable cardiac, neurological or gastrointestinal tract pathologies. Specifically, Chagas disease patients can exhibit various cardiac pathologies in the chronic stage of the infection, including myocarditis, myocardial hypertrophy, vasculitis and fibrosis, ultimately leading to heart failure [6,9]. Chagas disease remains the world’s leading cause of infectious myocarditis in afflicted individuals [10]. Chagasic cardiomyopathy’s severe health consequences are accompanied by a significant economic burden [8]. Several studies using ex vivo and in vitro murine cardiomyocyte culture models suggest that the parasite increases the expression of extracellular matrix components, which could lead to remodeling of the heart [11,12,13]. Studies using cardiac tissues from patients presenting with Chagasic cardiomyopathy suggest that genes associated with dilated cardiomyopathy and inflammatory responses play a significant role in the onset of Chagasic cardiac hypertrophy [14]. Additionally, studies have shown that *T. cruzi* can upregulate the expression of genes involved in extracellular matrix function and mitochondrial energy metabolism, which play a role in the development of cardiac hypertrophy [15,16]. Using primary human cardiomyocytes (PHCMs), our laboratory demonstrated that *T. cruzi* induced differential expression of fibrogenic genes during the early phase of infection; these genes include JunB, FOS, EGR1, EGR3 and SNAI1 [15]. We also showed that *T. cruzi* induced the expression of host thrombospondin-1 (TSP-1) during the early phase of infection [17,18]. TSP-1 has been reported to play a role in the regulation of fibrotic disorders through the activation of TGF-β, a profibrotic cytokine [19,20]. Others showed that active TGF-β is very important during *T. cruzi* infection [21]. *T. cruzi* has been reported to dysregulate calcium homeostasis to facilitate cellular infection [22]. Calcium binds to calmodulin (CaM) and activates the phosphatase calcineurin and CaM Kinase II [23]. Furthermore, it was suggested that *T. cruzi* infection induces an increase in endothelin-1 (ET-1) expression, which in turn plays an important role in the activation of calcineurin [24]. Activated calcineurin dephosphorylates cytoplasmic nuclear factor of activated T cells (NFATC), inducing its translocation into the nucleus, where it activates antiapoptotic and hypertrophic target genes [23,25,26,27]. However, the molecular mechanisms by which the parasite dysregulates gene expression in heart cells, especially during the early phase of cellular infection, remain elusive. Reports in the literature suggest that RNA-mediated gene silencing achieved via three small non-coding RNA (sncRNA) molecules—microRNAs (miRNAs), small interfering RNAs (siRNAs), and P-element induced wimpy testis (PIWI)-interacting RNAs (piRNAs)—play important roles in regulating gene expression by forming complexes with Argonaute proteins to recognize specific target sequences [28]. Although piRNAs were previously thought to function only in maintaining genome stability and integrity within the germline by silencing transposable elements (TE) [29], they are now being considered to play important roles in regulating gene expression [30,31,32]. TEs can be divided into two major classes. Class 1 (retrotransposons) is composed of long terminal repeat (LTR) retrotransposons and non-LTR retrotransposons, which include long and short interspersed nuclear elements (LINEs and SINEs), respectively. Class 2 elements (DNA transposons) also include subclasses based on the mechanism of chromosomal integration [33]. Approximately 45% of the mammalian genome is composed of retrotransposons which are known to play vital roles in neuronal cells, germ cells, somatic cells and diseases like diabetes, cancer and heart disease among others [31,34]. TEs have long been found in the genome, where the cells coopt TE sequences for cellular processes [35]. piRNAs have been classified as silencing sncRNAs derived from small silencing RNAs (ssRNA) [36,37]. Some studies suggested that piRNAs may regulate gene transcription [36]. This gene regulatory mechanism is thought to be similar to that of miRNAs [38].

Since piRNAs play essential roles in maintaining genomic stability and diversity, their dysregulation could lead to mutagenesis and chromosomal rearrangement, causing genetic mutations and diseases, including cancer [39,40]. Several piRNAs have been identified as biomarkers or potential targets in various cancers, including gastric cancer, hepatocellular carcinoma and colorectal cancer, even though their complete scope of functions is still being discovered [41,42,43,44]. There are conflicting schools of thought about the function of piRNAs in immune regulation. For example, tRNA-derived piRNAs expressed in monocytes can function as mediators of interleukin-4 (IL-4) expression in immune cells to regulate the immune response [45]. It was suggested that piRNA-mediated RNA interference (RNAi) potentiates an antiviral response in mosquito cells [46] while another study concluded that the piRNA pathway is not necessary for antiviral defense in *Drosophila* [47].

In heart cells, piRNAs regulate AKT signaling through interaction with the PIWIL2 protein, thereby playing an important role in cardiomyocyte proliferation and regeneration [31,32]. Patients presenting with myocardial infarction have a significantly elevated level of piR-2106027, which has been suggested to be an important diagnostic marker for the disease [32,48]. Despite all these important functions of known piRNAs, the molecular signature of the *T. cruzi*-induced piRNA profile in human cardiomyocytes during the early phase of infection remains unknown. Here, we challenged PHCM with *T. cruzi* and evaluated the piRNA expression profile (piRNome kinetics) during the early phase of infection. We show that *T. cruzi* dysregulated the piRNA expression profile in PHCM during the early phase of infection. We also observed that during the early phase of infection, *T. cruzi* induced the differential expression of currently unreported putative piRNAs (novel piRNAs). In silico analysis using miRanda and RNA22 showed that the novel piRNAs mapped to specific target regions in genes coding for NFATC2, FOS and TGF-β1 reported to be important during the early phase of *T. cruzi* infection. We built a framework that links known and putative novel piRNAs to their potential molecular targets during the early infection process. Furthermore, we connect them in biological networks to theoretically determine piRNA pathway-level interactions involved in *T. cruzi* pathogenesis. This novel finding showing that a pathogen can dysregulate host piRNA expression can be exploited for the development of molecular intervention strategies during the early phase of *T. cruzi* infection.

## 2. Results

### 2.1. piRNAs are Differentially Expressed in PHCM during the Early Phase of T. cruzi Infection

**Figure 1:**
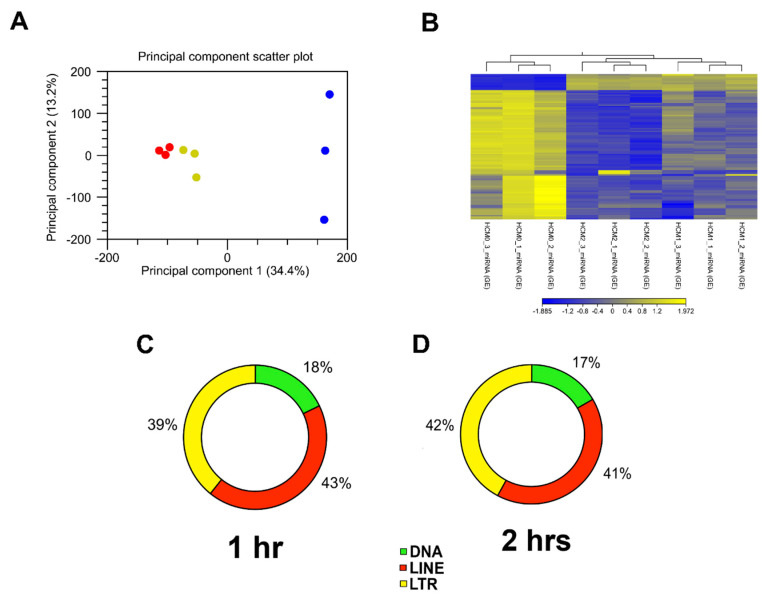
Analysis of RNA-seq data and putative piRNAs induced by *T. cruzi* mapped to different transposable element subfamilies. (**A**) Principal component analysis (PCA) of gene expression was performed for all samples and all probe sets, by using a median centering of the data set. The *x*-axis corresponds to principal component 1 (PC1) and the *y*-axis to the principal component 2 (PC2); the percentage of the variance is indicated between brackets. Based on their small non-coding RNA expression values, samples from each time point (0, 1 and 2 h) clustered together, confirming homogeneity of the gene expression profiles within each group. The group of *T. cruzi* challenged samples clustered independently of the control group (blue = 0 h, yellow = 1 h, red = 2 h). (**B**) Heatmap and hierarchical clustering was performed on control and test samples, 0, 1 and 2 h, respectively, using Euclidean distance measure and single linkage analysis. Each column represents one sample and each row represents one non-small coding RNA within the data set. The color-coded scale illustrating the relative expression after global normalization is indicated. Differentially expressed putative and known piRNAs induced post parasite challenge of PHCM at (**C**) 1 h and (**D**) 2 h, respectively, belong to different transposable element families.

To evaluate whether *T. cruzi* can dysregulate the expression of piRNAs in heart cells, we challenged PHCM with invasive *T. cruzi* trypomastigotes Tulahuen strain, clone MMC20A. Purified sncRNA were ligated, reverse transcribed and amplified by PCR. DNA-Nanoballs (DNB) generated were subjected to RNAseq using the BGISEQ-500 sequencing platform. To analyze the clustering of the replicates and their variance, we carried out a principle component analysis (PCA) of all sequenced sncRNAs, which also include piRNAs. The analytical output showed that the biological replicates of the time points in the experiment segregated independently of each other, as shown in the principal component scatter plot. Principal component 1 (34.4% variance) and principal component 2 (13.2% variance) showed that the samples from each group clustered together (Figure 1A). Furthermore, hierarchical clustering, which also evaluates whether the gene expression profiles of sncRNAs at each time point were specific, showed that replicates from each time point grouped together (Figure 1B). The one- and two-hour cluster replicates are separate from the control replicate cluster, indicating that the differential expression changes are substantial. Since piRNAs are generated from transposable element (TE) sequences to target and repress mobile genetic elements, it would therefore be anticipated that piRNAs can also be classified into TE families. Mapping of the differentially expressed piRNA sequences to their genomic loci leads to their classification as DNA, LINE and LTR TE families. A large proportion of the differentially expressed piRNAs originated from functionally active LINE retrotransposons, representing 43% at the 1 h and 41% at 2 h time points (Figure 1C,D).

### 2.2. T. cruzi Induces Differential piRNA Expression in PHCMs

**Figure 2:**
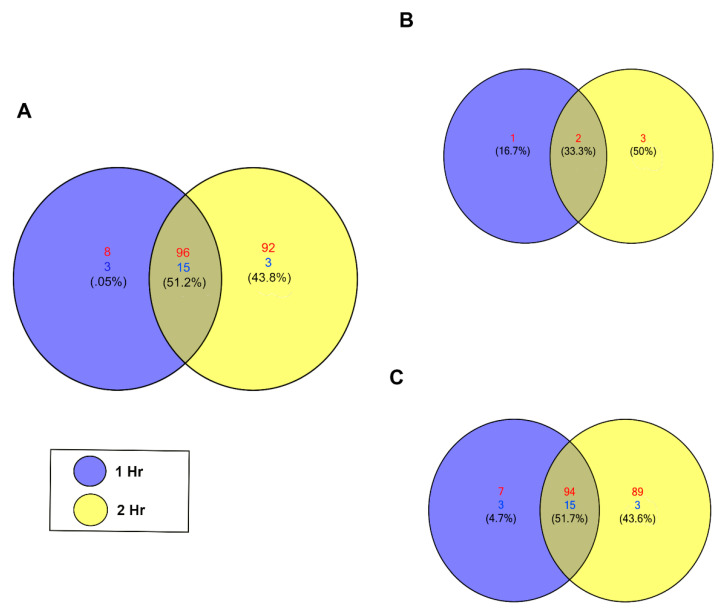
*T. cruzi* induces differential expression of piRNAs in PHCM. (**A**) Venn diagram of upregulated and downregulated piRNAs counts at different time points after *T. cruzi* challenge of PHCM, FDR corrected *p* < 0.05. (**B**) Venn diagram generated showing the number of differentially expressed known piRNAs at 1 and 2 h post parasite challenge of PHCM. These previously reported and annotated piRNAs are available in the NCBI database. (**C**) Venn diagram showing distribution of putative piRNAs that were differentially expressed in PHCM at different time points after *T. cruzi* challenge. These piRNAs which were not previously published have now been deposited in the NCBI database.

To detect significant changes in the piRNA expression profile in PHCMs during the early phase of *T. cruzi* infection, we performed differential expression analysis using NOIseq [49]. We identified 217 unique piRNAs that were significantly differentially expressed (NOIseq probability of differential expression, q ≥ 0.8). Among these, most were upregulated, with a minimum count of 104 unique piRNAs at the 1 h time point, which increased to a maximum count of 188 unique piRNAs at the 2 h time point. At both time points, 18 piRNAs were downregulated (Figure 2A). Supplemental Tables S1 and S2 show the statistical values and sequence for each piRNA. Known piRNAs accounted for a small portion of the differentially expressed piRNAs. Only two known piRNAs were substantially differentially expressed at both time points (Figure 2B) among all six of these piRNAs that were upregulated between the two time points. The majority of the significantly differentially expressed piRNAs (q ≥ 0.8) are putative piRNAs; 94 were upregulated and 15 downregulated at both time points (Figure 2C). Putative piRNAs accounted for all downregulated piRNAs. More piRNAs were differentially expressed at the 2 h time point compared to the 1 h time point.

### 2.3. Differentially Expressed piRNAs Predicted to Target Genes Associated with Early Phase of T. cruzi Infection

**Figure 3:**
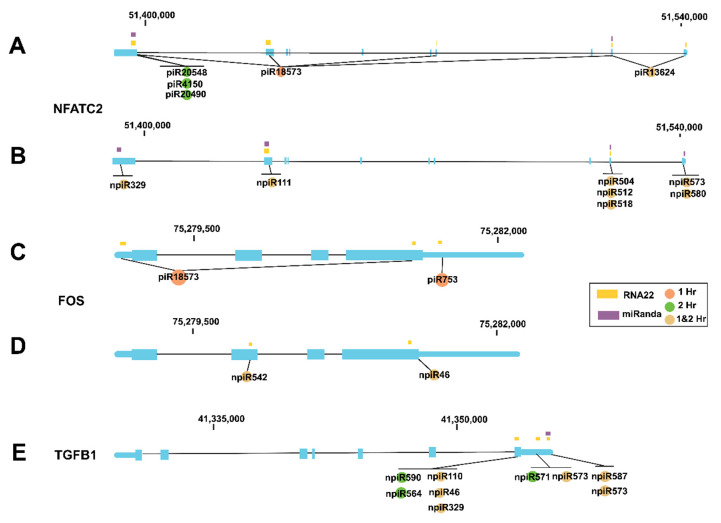
piRNAs differentially expressed in PHCM during the early phase of *T. cruzi* infection mapped to specific genetic regions. miRANDA and RNA22 algorithms were used to predict target binding sites of known and novel piRNAs. In the genetic cartoons, exons represented by blocks are connected by horizontal lines representing introns while the 5′ and 3′ untranslated regions (UTRs) are represented as thinner blocks at the extremities of each gene, respectively. NFATC2 gene cartoons (3′ to 5′ orientation) showing positions where (**A**) known piRNAs and (**B**) novel piRNAs that are differentially expressed at 1, 2 h or both time points are predicted to be mapped, respectively. Cartoons of FOS gene (5′–3′) showing positions where (**C**) known piRNAs and (**D**) novel piRNAs that are differentially expressed at 1, 2 h or both time points are predicted to be mapped, respectively. (**E**) Cartoon of TGF-β1 gene (3′–5′) showing positions where differentially expressed novel piRNAs are mapped.

We and others showed that during the early phase of cellular infection by *T. cruzi*, the expression profiles of AP-1 transcription factor network genes including NFATC2, FOS and TGF-β are dysregulated by the parasite. To evaluate whether some of the piRNAs (known and novel) that were induced by the parasite can potentially target any of those previously reported genes, we used miRANDA and RNA22 algorithms [50] to predict target binding sites. The resulting 425 predicted target genes were then mapped to KEGG pathways, indicating pathway-level enrichment of the focal adhesion (*p* = 0.00015497, FDR q = 0.040156), regulation of actin cytoskeleton (*p* = 0.00028087, FDR q = 0.040156) and MAPK signaling pathway (*p* = 0.00036953, FDR q = 0.040156). The mitogen-activated protein kinase (MAPK) pathway has been extensively linked to the stimulation of AP-1 transcription factors in *T. cruzi*-induced cardiomyopathy [51,52]. Cardiac tissue stress triggers activation of the MAPK signal cascade, leading to increased activity of extracellular signal-regulated kinase (ERK) and Janus kinase (JNK) [53]. Subsequent activation of AP-1 transcription factors upregulates endothelin-1, ultimately leading to increased expression of inflammatory cytokines, driving cardiomyopathy [54]. Our in silico analysis showed that both the known and novel piRNAs have target sites on all of the AP-1 genes that we and others reported to be dysregulated by the parasite during the early phase of cellular infection (Figure 3A–E). Five known piRNAs have target sites on NFATC2 and seven novel piRNAs have potential target sites on NFATC2 (Figure 3A,B). Two known piRNAs differentially expressed at 1 h target FOS while two novel piRNAs differentially expressed at both time points target FOS (Figure 3C,D). Nine novel differentially expressed piRNAs target the TGF-β1 gene, six of them at both time points (Figure 3E).

### 2.4. Biological Interaction Network of Differentially Expressed piRNA and AP-1 Transcription Factors during the Early Phase of T. cruzi Infection

**Figure 4:**
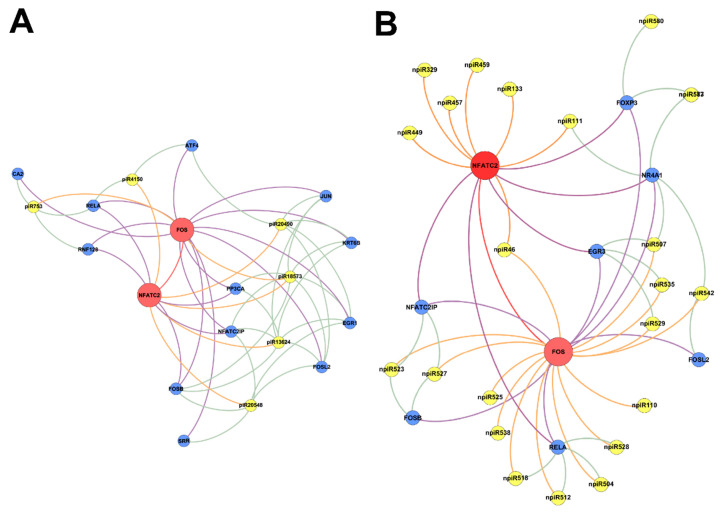
Networks of differentially expressed piRNAs targeting FOS and NFATC2 operating during PHCM challenge with *T. cruzi.* Biological interaction networks were created using predicted piRNA target genes (FOS, NFATC2) as primary seed nodes to query pathway and interaction data sources out to one degree of interaction. Primary seed nodes are displayed as red circles. Expansion nodes (blue circles) with a predicted binding target of a differentially expressed known or novel piRNA (yellow node) were added to the network. Connections between nodes (edges) represent interactions between the different biological entities. Networks of (**A**) known piRNAs and (**B**) novel piRNAs targeting FOS, NFATC2 and adjacent genes from expansion to one degree of interaction during all time points.

**Figure 5:**
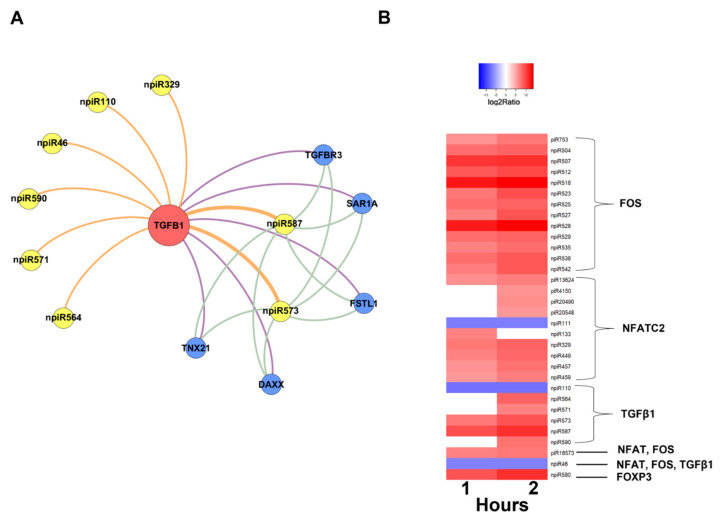
Network of differentially expressed novel piRNAs targeting TGF-β1 and heatmap of differentially expressed piRNAs in PHCM induced early during *T. cruzi* infection. Intensity plot of differentially expressed piRNAs, having greater than two-fold up- or downregulation (log2 ratio) with a probability of differential expression q-value ≥ 0.8. Color-coded scale illustrates the normalized relative expression. (**A**) Network of novel piRNAs targeting TGF-β1 (seed node) and adjacent genes to one degree of interaction at all time points. (**B**) Heatmap of differentially expressed known and novel piRNAs targeting NFATC2, FOS and TGF-β1 during the early infection phase.

In order to understand the molecular mechanisms that could be triggered in PHCM by piRNAs induced during the early phase of cellular infection by *T. cruzi*, we used the GeneMANIA algorithm to construct biological interaction networks connecting known piRNAs to FOS and NFATC2 expanded to one degree of molecular protein–protein interactions and visualized with Gephi (Figure 4A). We show that the known piRNAs that were differentially induced can potentially target not only FOS and NFATC2 but also proteins that have the potential to interact with them. Additionally, in a network similarly generated using the putative novel piRNAs that were also significantly differentially expressed, two-fold up- or downregulation (log2 ratio) with a probability of differential expression q-value ≥ 0.8 was generated (Figure 4B). The novel piRNAs have predicted binding sites on the AP-1 transcription factors and proteins that were expanded to one degree of interaction. The early phase of *T. cruzi* infection significantly induced the expression of novel piRNAs, which we used in the generation of the network (Figure 5A). We show that two of the differentially expressed novel piRNAs can target all the one degree of interaction proteins which include TGFβ Receptor 3 (TGFBR3). The heatmap of the substantially differentially expressed known and novel piRNAs that are mapped to target the AP-1 transcription factors focused on in this report is shown in Figure 5B.

## 3. Discussion

*T. cruzi*, the causative agent of Chagasic heart disease, is a new global health threat which is now common in North America [1,2,3,4,5,6,55]. The molecular mechanisms by which *T. cruzi* alters the gene expression profile in human heart cells leading to fibrogenic pathologic responses are still largely unknown. Invasive *T. cruzi* trypomastigotes can invade endothelial cells and vascular smooth muscle cells and penetrate interstitial vasculature to eventually invade cardiomyocytes. The invasion and destruction of cardiomyocytes leads to conductive abnormalities and ensuing pathology [6]. Primary human cardiomyocytes are suitable for studying the mechanism by which *T. cruzi* causes pathologic changes in the transcriptome and physiology of the heart. In a previous study, we challenged PHCM with *T. cruzi* and evaluated the gene expression profile by microarray analysis to show that the parasite induced significant differential expression of many fibrogenic genes including the AP-1 transcription factor network [15]. Ongoing research shows that microarray studies have shortcomings that can be overcome with contemporary RNA-sequencing technology [56]. In this study, we hypothesize that during the early phase of infection, *T. cruzi* dysregulates the expression of gene regulatory molecules in PHCM, including piRNAs. This dysregulation potentially alters the expression of fibrogenic genes that we and others have reported previously [15,24]. piRNAs have been shown to play an important role in the regulation of the AKT pathway in heart cells, indicating that the piRNA profile is important in cardiac homeostasis.

To evaluate the differential expression of piRNAs (piRNome) during the early phase of *T. cruzi* infection of PHCM, we purified sncRNA from *T. cruzi* challenged PHCM for RNAseq analysis. We used the combinatorial probe-anchor synthesis (cPAS)-based BGISEQ-500 sequencing platform that combines template enrichment using rolling circle amplification on DNB nanoarrays followed by stepwise sequencing using polymerase. This approach is important because it applied linear DNA amplification instead of exponential DNA amplification to make sequencing arrays, resulting in lower error accumulation and sequencing bias, thereby improving the quality of our data [57,58].

Here, we show that the pathogen, *T. cruzi*, dysregulates the expression pattern of piRNAs in parasite challenged PHCM. Before in-depth analysis, we conducted an NCBI BLAST search of the putative piRNA sequences against the *T. cruzi* sequence database. We observed no significant alignments, suggesting that the piRNAs are host-derived. Our bioinformatics analysis of the differentially expressed piRNAs showed that piRNA experimental groups segregated independently of each other, the control replicates clustering away from challenged PHCM replicates indicating that the differential expression changes are substantial (Figure 1A,B). The data show a time-dependent piRNA expression profile in PHCM in response to *T. cruzi* challenge. Many piRNAs are generated from TE, which were hitherto classified as a group of “junk DNA” in the mammalian genome with unknown functions. The generated piRNAs target and silence TE mRNAs, indicating that the piRNAs can be classified into TE families, class 1 including long interspersed elements (LINE) and long terminal repeats (LTR) and class 2 including DNA transposons [33,59]. The differentially expressed piRNAs, both known and novel, reported in our study were classified into the TE subfamilies (Figure 1C,D). Of all the dysregulated piRNAs, ~43% of them arise from LINEs after 1 h compared to 41% at 2 h. The proportion of piRNAs arising from LTRs increased continuously from 39% at the 1 h time point to 42% at the 2 h time point. This increasing percentage of dysregulated piRNAs originating from LTRs is noteworthy. LTR regions typically contain promoters, and the insertion of mobile elements in these regions could play a significant role in regulating the transcription of the neighboring genes or the piRNAs themselves [60,61]. Thus, the high percentage of piRNAs originating from LTRs suggests an important role in the regulation of gene expression during the early phase of *T. cruzi* infection.

More than 75% of the cumulative differentially expressed piRNAs induced by *T. cruzi* in PHCM arise from LTRs and LINEs, suggesting that these regions are the primary source of piRNAs during the early phase of *T. cruzi* infection compared to the piRNAs arising from DNA elements. Our data show that during the early phase of *T. cruzi* infection of PHCM, the parasite induced significant upregulation/downregulation of different piRNAs (Figure 2). This suggests that the parasite dysregulates the host gene regulatory system by significantly altering the differential expression of different piRNAs. The majority of the differentially expressed piRNAs that we report here are putative (novel) piRNAs. These novel piRNAs exhibit characteristics of previously reported canonical piRNAs [31,62,63].

Previous studies showed that seed sequences are necessary for predicting piRNA–mRNA binding, which suggests that piRNA targeting mimics that of miRNAs [38].

Due to the overlapping seed sequence positions of both piRNAs and miRNAs, we adapted miRanda and RNA22 algorithms originally designed for predicting miRNA targeting, as shown by others in our bioinformatic analysis [64].

Differentially expressed piRNAs and their predicted targets, NFATC2, FOS and TGF-β1, were used to generate networks that linked the piRNAs to their target genes and expanded them to one degree of freedom. In silico analysis showed that some of the differentially expressed piRNAs (known and novel) can target genes that we and others reported to be dysregulated during the early phase of *T. cruzi* infection (Figure 3, Figure 4 and Figure 5A). The piRNAs were also predicted to target sequences on genes that were within one degree of interaction with the AP-1 transcription factors evaluated in this study (Figure 4 and Figure 5A). We also show that a novel piRNA (npiR46) can bind to target sequences on both NFATC2 and FOS (Figure 4B). Our results show that novel piRNAs bind to target sequences on TGFβ1 and only two novel piRNAs (npiR573 and 587) target genes with one degree of interaction to TGFβ1 (Figure 5B). It has been suggested that piRNAs can regulate gene expression in a variety of ways [65], but the exact molecular mechanisms by which these piRNAs regulate the genes reported in this study remain elusive and are under investigation in our laboratory. It is also not known if a threshold number of piRNAs is required for the regulation of gene expression reported during *T. cruzi* infection. Various reports have proposed piRNAs as biomarkers in cancer and predictors of the pathology of colorectal and ovarian cancers [63,66]. However, the piRNA signature or the exact role that these differentially regulated piRNAs play in the pathogenesis of *T. cruzi* infection remains largely unknown. Taken together, our data show that during the early phase of infection, *T. cruzi* trypomastigotes dysregulate and thereby cause significant differential expression of piRNAs in PHCM. These *T. cruzi*-induced piRNAs may serve as important biomarkers for the early phase of *T. cruzi* infection. This is the first report showing that a pathogen can dysregulate host piRNA expression, potentially leading to enhanced understanding of parasite-induced gene regulation, which is important for the development of molecular intervention strategies during the early phase of *T. cruzi* infection.

## 4. Materials and Methods

### 4.1. Primary Human Cardiomyocyte Culture

Primary human cardiac myocytes (PHCM) were obtained from and cultured following the manufacturer’s recommendations (PromoCell, Heidelberg, Germany). Briefly, the PHCM were cultured in myocyte basal growth medium supplemented with the supplemental mix (PromoCell, Heidelberg, Germany) containing fetal calf serum (0.05 mL/mL), recombinant human epidermal growth factor (0.5 ng/mL), recombinant human basic fibroblast growth factor (2 ng/mL) and recombinant human insulin (5 ug/mL). The cells were cultured in T75 flasks at 37 °C in the presence of 5% CO_2_ to approximately 80% confluency (approximately 4 × 10^6^ cells) prior to being used in our assays.

### 4.2. Parasite Culture and Infection Assays

Heart myoblast monolayers at 80% confluence, cultured in complete DMEM containing 5% glutamax, 10% fetal bovine serum, 1% each of penicillin/streptomycin, multivitamins and MEM non-essential amino acids (Life Technologies, Carlsbad, CA, USA), were infected with *T. cruzi* trypomastigotes. Pure cultures of highly invasive *T. cruzi* trypomastigotes (clone MMC 20A, Tulahuen strain) were harvested from the supernatant of infected heart myoblast monolayers as previously described [67,68]. The parasites were washed with Hanks Balanced Salt Solution (HBSS) and resuspended in PHCM growth medium without supplement at 1 × 10^7^ parasites/mL. For the infection assays, confluent PHCM monolayers were starved in HBSS containing 30 mM HEPES, followed by the addition of *T. cruzi* trypomastigotes in PHCM growth medium without supplements at a ratio of 10 parasites per cell. Parasite-challenged PHCMs were incubated for 1 and 2 h, respectively, in triplicate. Total and small RNAs were extracted from all samples. Mock-infected (media only) PHCMs served as control.

### 4.3. RNA Extraction and Quality Assessment

Control and parasite-challenged PHCM were washed with HBSS. The cells were lysed in QIAzol, an RNA extraction lysis buffer and extracted with chloroform following the manufacturer’s instructions (Qiagen, Valencia, CA, USA). The aqueous phase of the extract was mixed with an equal volume of 70% ethanol and passed through the RNeasy Mini spin column. The eluate, which contained small RNA species, was mixed with 0.65 volumes of pure ethanol and passed through an RNeasy MiniElute spin column. The column was washed with 80% ethanol and bound small RNA species were eluted with RNase-free water essentially as described by the manufacturer (Qiagen). Large RNAs bound to the RNeasy mini spin column were washed and eluted with RNase-free water as described by the manufacturer (Qiagen). The quality of the purified RNA was analyzed using the Bioanalyzer 2100 system (Agilent Technologies, Santa Clara, CA, USA) to determine the RNA Integrity Number (RIN). Samples with a RIN of at least 8 were considered for further analysis.

### 4.4. RNA-Sequencing of sncRNA, Filtering and Expression Evaluation

Briefly, purified small RNA species ranging from 18 to 30 nt were ligated to the Illumina 3′ adaptor and 5′ adaptor. Ligation products were gel-purified, reverse-transcribed and amplified by Rolling-Circle Replication (RCR). This linear amplification only copies the original DNA template instead of copy-of-a-copy in order to make small, very high-density sequencing compacted templates called DNA NanoBalls (DNBs). The DNBs were compacted on high-density patterned nanoarray and sequenced by combinatorial Probe-Anchor Synthesis (cPAS), a sequencing chemistry technique which is optimized for DNBseq. The combination of linear amplification and DNB technology reduces the error rate while enhancing the signal. Smaller DNB spots with highly concentrated DNA deliver significantly higher dye density than PCR cluster arrays, leading to higher signal-to-noise for optimal imaging signal integrity. High-throughput sequencing (HTS) was conducted using the BGISEQ-500 sequencing platform for small RNA (BGI, Cambridge, MA, USA) utilizing the NGS 2.0 DNBseq technology. The DNBseq sequencing technology (NGS 2.0) combines the power of DNA Nanoballs (DNB), PCR-free Rolling Circle Replication, Patterned Nano Arrays and cPAS to deliver a new level of data clarity and reliability. The BGISEQ-500 sequencing platform has comparable sensitivity and accuracy in terms of quantification of gene expression, and low technical variability as compared to the Illumina HiSeq platform [69,70]. High-throughput sequencing (HTS) was conducted using the BGISEQ-500 sequencing platform for small RNA (BGI, Cambridge, MA, USA) [57,71]. In total, 30,001,700 raw HTS data were filtered by eliminating low-quality reads (less than 20 in Phred quality score), as well as removing confounders such as adaptors and other contaminants including missing 3′ primers, 5′ primer contamination and incomplete small non-coding RNA (sncRNA) reads of less than 18 bp. Reads that met our analysis criteria were subjected to size filtration for piRNA to select for sncRNA transcripts that were 25–30 bp in length and did not match any known miRNA or siRNA sequences. Bowtie was used to map the reads to reference genomes [72]. The piRNA annotation program (Piano) was used to predict known piRNAs via a support vector machine (SVM) algorithm with transposon interaction informatics [73]. piRNA expression was determined using the standard transcripts per kilobase million mapped (TPM) method [74].

### 4.5. Analysis of Differential piRNA Expression

The NOISeq method (version 3.34) was used to determine differentially expressed piRNAs. Each time point was screened for significantly different piRNA expression compared to control by calculating the log_2_ fold-change (M) and absolute differential value (D) between each pair of time points to build a noise distribution model. A piRNA is counted as differentially expressed if M and D values are likely to be higher than in noise. The significance threshold for differential piRNA expression was set to fold-change ≥2 or ≤2 and a divergence probability ≥0.8. A probability of 0.8 is equivalent to an odds value of 4:1, meaning that a given piRNA is 4 times more likely to be differentially expressed than non-differentially expressed [49].

### 4.6. piRNA Target Prediction

Differentially expressed piRNAs were compared against hg38 RefSeq transcripts in miRanda [50] using a high-stringency pairing score cutoff of ≥175, an energy cutoff of ≤−30 kcal/mol and a requirement for exact seed region alignment. The prediction was also made in RNA22 [75] using a high specificity setting.

### 4.7. Mapping of Biological Pathway Interactions

Pathway enrichment analysis was performed with WEBGESTALT web analysis software [76,77] (http://bioinfo.vanderbilt.edu/wg2/) by mapping predicted piRNA targets to corresponding enriched KEGG [78] (https://www.genome.jp/kegg/) pathways and conducting overrepresentation analysis. Significance for pathway-level enrichment was defined as having an enrichment score false discovery rate (FDR) corrected *p*-value < 0.05. Biological interaction network construction was conducted with the GeneMANIA algorithm [76,77] by querying multiple biological interaction databases including GEO, BioGRID and EMBL-EBI. Predicted piRNA targets connected to the AP-1 transcription factor family were set as starting seed nodes, and the network was then expanded to one degree of biological interaction using GeneMANIA and visualized with Gephi [79] (https://gephi.org/).

### 4.8. Data Availability

All relevant data not presented in the manuscript are located in the SRA database: SRA accession: PRJNA635217 (https://www.ncbi.nlm.nih.gov/sra/PRJNA635217).

## Supplementary Materials

Supplementary Materials can be found at https://www.mdpi.com/1422-0067/21/24/9439/s1.

## Abbreviations

DMEM	Dulbecco’s modified eagle medium
FDR	False discovery rate
PHCM	Primary human cardiac myocytes
piRNA	PIWI-interacting RNAs
snRNA	Small-noncoding RNAs
*T. cruzi*	*Trypanosoma cruzi*
