# Supplementary material for: Trypanosoma cruzi Modulates PIWI-Interacting RNA Expression in Primary Human Cardiac Myocytes during the Early Phase of Infection
Source: Int J Mol Sci. 2020 Dec 11;21(24):9439. doi: 10.3390/ijms21249439 (PMC7764157; doi:10.3390/ijms21249439)
Supplement: Supplementary file 1 [file ijms-21-09439-s001.pdf]

**Supplementary Table S1.** Differential expression values for piRNAs at 1 hour and 2 hours.

| piRNA id | Mean expression (Control) | Mean expression (1hr) | Mean expression (2hr) | log2Ratio (1hr) | Probability (q,1hr) | log2Ratio(2hr) | Probability(q,2hr) |
|---|---|---|---|---|---|---|---|
| novel_pir969 | 0.01 | 6.753333 | 10.54333 | 9.399456 | 0.947358 | 10.04212 | 0.966732 |
| novel_pir535 | 0.01 | 1.143333 | 2.176667 | 6.837102 | 0.825203 | 7.765977 | 0.895956 |
| hsa_piR_013624 | 0.213333 | 14.93 | 29.31 | 6.128961 | 0.876016 | 7.10214 | 0.954664 |
| novel_pir1628 | 0.01 | 5.073333 | 11.48333 | 8.98679 | 0.935569 | 10.16533 | 0.968037 |
| novel_pir1027 | 0.01 | 4.16 | 9.166667 | 8.70044 | 0.92378 | 9.840253 | 0.963307 |
| novel_pir1391 | 0.01 | 5.29 | 9.89 | 9.047124 | 0.939024 | 9.949827 | 0.96559 |
| novel_pir952 | 0.01 | 1.836667 | 3.643333 | 7.520946 | 0.871138 | 8.509115 | 0.927919 |
| novel_pir976 | 0.01 | 18.07333 | 71.97 | 10.81965 | 0.975203 | 12.81318 | 0.996249 |
| novel_pir1411 | 0.01 | 1.17 | 2.503333 | 6.870365 | 0.827439 | 7.967707 | 0.905414 |
| novel_pir1028 | 0.01 | 7.403333 | 17.32667 | 9.532031 | 0.95122 | 10.75878 | 0.977821 |
| novel_pir905 | 1.373333 | 0.01 | 0.01 | -7.10154 | 0.841667 | -7.10154 | 0.854044 |
| novel_pir518 | 0.01 | 56.33333 | 116.66 | 12.45977 | 0.992276 | 13.51002 | 0.997554 |
| novel_pir512 | 0.01 | 5.013333 | 8.58 | 8.969626 | 0.934756 | 9.744834 | 0.962166 |
| novel_pir797 | 0.01 | 1.12 | NA | 6.807355 | 0.823374 | NA | NA |
| novel_pir975 | 0.01 | 2.716667 | 3.863333 | 8.085694 | 0.900203 | 8.593702 | 0.93167 |
| novel_pir996 | 0.01 | 1.553333 | 2 | 7.279224 | 0.854472 | 7.643856 | 0.890411 |
| novel_pir984 | 0.383333 | 139.87 | 291.2533 | 8.511271 | 0.983333 | 9.569459 | 0.997717 |
| novel_pir1686 | 0.01 | 1.006667 | 1.54 | 6.653442 | 0.809959 | 7.266787 | 0.863666 |
| novel_pir1246 | 1.53 | 0.01 | 0.01 | -7.25739 | 0.853252 | -7.25739 | 0.863177 |
| novel_pir960 | 0.01 | 6.246667 | 16.11333 | 9.286943 | 0.945325 | 10.65404 | 0.977006 |
| novel_pir1117 | 0.01 | 2.12 | 3.073333 | 7.72792 | 0.88374 | 8.26366 | 0.919113 |
| novel_pir1324 | 0.01 | 1.16 | 1.636667 | 6.857981 | 0.827033 | 7.354617 | 0.870841 |
| novel_pir1627 | 0.01 | 23.90667 | 38.14333 | 11.2232 | 0.981098 | 11.89722 | 0.9894 |
| novel_pir187 | 1.716667 | 0.01 | 0.01 | -7.42347 | 0.864431 | -7.42347 | 0.875408 |
| novel_pir457 | 0.656667 | 40.60333 | 111.7633 | 5.950293 | 0.880488 | 7.41107 | 0.975701 |
| novel_pir970 | 0.01 | 2.2 | 2.27 | 7.78136 | 0.885569 | 7.826548 | 0.89938 |
| novel_pir1575 | 0.01 | 1.686667 | 3.846667 | 7.398031 | 0.862805 | 8.587465 | 0.931507 |
| novel_pir998 | 0.01 | 1.736667 | 2.41 | 7.440177 | 0.865447 | 7.912889 | 0.9015 |
| novel_pir935 | 0.01 | 1.69 | 2.906667 | 7.400879 | 0.862805 | 8.183222 | 0.916504 |
| novel_pir1231 | 0.01 | 7.56 | 21.81667 | 9.562242 | 0.952642 | 11.09121 | 0.981572 |
| novel_pir971 | 0.01 | 1.263333 | 3.12 | 6.981092 | 0.833537 | 8.285402 | 0.920091 |
| novel_pir1703 | 0.01 | 12.55667 | 24.85667 | 10.29424 | 0.967276 | 11.27942 | 0.983692 |
| novel_pir128 | 0.01 | 1.126667 | NA | 6.815917 | 0.825 | NA | NA |
| novel_pir957 | 0.01 | 1.143333 | 1.58 | 6.837102 | 0.825203 | 7.303781 | 0.867091 |
| novel_pir1259 | 0.01 | 1.716667 | 1.583333 | 7.423466 | 0.864431 | 7.306821 | 0.867091 |
| hsa_piR_000753 | 0.313333 | 16.97667 | NA | 5.759711 | 0.831504 | NA | NA |

| | | | | | | | |
|---|---|---|---|---|---|---|---|
| novel_pir772 | 1.816667 | 0.01 | 0.01 | -7.50515 | 0.870325 | -7.50515 | 0.880952 |
| novel_pir981 | 0.15 | 9.193333 | 15.90667 | 5.937554 | 0.852642 | 6.728525 | 0.931507 |
| novel_pir982 | 0.01 | 2.24 | 4.403333 | 7.807355 | 0.886789 | 8.782452 | 0.938193 |
| novel_pir329 | 0.01 | 1.57 | 3.276667 | 7.294621 | 0.856504 | 8.356085 | 0.92319 |
| novel_pir525 | 0.166667 | 36.23 | 59.76667 | 7.764075 | 0.965854 | 8.486232 | 0.985812 |
| novel_pir1004 | 0.01 | 4.04 | 3.18 | 8.658211 | 0.922154 | 8.312883 | 0.920581 |
| novel_pir972 | 0.01 | 1.936667 | 3.553333 | 7.597432 | 0.878049 | 8.473029 | 0.926778 |
| novel_pir1122 | 0.01 | 3.216667 | 2.886667 | 8.329423 | 0.910163 | 8.173261 | 0.916014 |
| novel_pir528 | 0.01 | 49.67333 | 132.62 | 12.27826 | 0.99126 | 13.69501 | 0.99788 |
| novel_pir1229 | 0.01 | 31.00667 | 78.88667 | 11.59836 | 0.984756 | 12.94557 | 0.996902 |
| novel_pir536 | 0.01 | 2.423333 | 3.113333 | 7.920849 | 0.89187 | 8.282316 | 0.920091 |
| novel_pir449 | 0.01 | 0.953333 | 2.656667 | 6.574909 | 0.802846 | 8.053473 | 0.909654 |
| novel_pir983 | 0.143333 | 20.37333 | 28.28 | 7.151164 | 0.943496 | 7.624264 | 0.968363 |
| novel_pir1023 | 0.01 | 7.116667 | 3.973333 | 9.475058 | 0.949797 | 8.634206 | 0.932648 |
| novel_pir1003 | 0.01 | 0.95 | 1.303333 | 6.569856 | 0.802846 | 7.026062 | 0.847358 |
| novel_pir459 | 0.493333 | 24.02333 | 57.13 | 5.60573 | 0.814431 | 6.855542 | 0.954664 |
| novel_pir1102 | 0.01 | 1.093333 | NA | 6.77259 | 0.822154 | NA | NA |
| novel_pir1685 | 0.01 | 1.54 | 2.776667 | 7.266787 | 0.853659 | 8.11721 | 0.912427 |
| novel_pir527 | 0.01 | 1.15 | 5.273333 | 6.84549 | 0.826626 | 9.042571 | 0.947652 |
| novel_pir607 | 0.01 | 1.186667 | 1.413333 | 6.890771 | 0.828659 | 7.142958 | 0.856001 |
| novel_pir256 | 0.01 | 1.403333 | NA | 7.132714 | 0.843902 | NA | NA |
| novel_pir519 | 0.01 | 1.383333 | 2.31 | 7.112005 | 0.842683 | 7.851749 | 0.900359 |
| hsa_piR_018573 | 0.2 | 20.57333 | 32.24333 | 6.684632 | 0.926829 | 7.332857 | 0.963796 |
| novel_pir1601 | 0.01 | 1.163333 | 1.82 | 6.862121 | 0.827033 | 7.507795 | 0.880952 |
| novel_pir580 | 0.01 | 6.23 | 20.42 | 9.283088 | 0.945325 | 10.99577 | 0.980431 |
| novel_pir1233 | 0.01 | 0.99 | 1.326667 | 6.629357 | 0.80874 | 7.051662 | 0.85013 |
| novel_pir955 | 0.156667 | 11.11 | 24.62 | 6.148017 | 0.872967 | 7.295989 | 0.95923 |
| novel_pir857 | 0.01 | 5.343333 | 4.243333 | 9.061596 | 0.939228 | 8.729054 | 0.936399 |
| novel_pir1002 | 0.01 | 1.8 | 3.23 | 7.491853 | 0.869715 | 8.33539 | 0.921559 |
| novel_pir1118 | 0.01 | 4.626667 | 4.306667 | 8.853829 | 0.931098 | 8.750428 | 0.937704 |
| novel_pir1264 | 0.143333 | 8.36 | 12.24667 | 5.866057 | 0.837602 | 6.416871 | 0.90835 |
| novel_pir111 | 1.386667 | 0.01 | 0.01 | -7.11548 | 0.843293 | -7.11548 | 0.854697 |
| novel_pir965 | 0.01 | 4.533333 | 6.253333 | 8.824428 | 0.928659 | 9.288482 | 0.952544 |
| novel_pir1315 | 0.01 | 1.703333 | 2.673333 | 7.412217 | 0.864024 | 8.062496 | 0.909817 |
| novel_pir1136 | 0.973333 | 0.01 | 0.01 | -6.60486 | 0.804675 | -6.60486 | 0.809035 |
| novel_pir1618 | 1.573333 | 0.01 | 0.01 | -7.29768 | 0.856504 | -7.29768 | 0.866601 |
| novel_pir1103 | 0.01 | 3.076667 | 2.06 | 8.265224 | 0.907724 | 7.686501 | 0.89302 |
| novel_pir999 | 0.01 | 3.206667 | 2.943333 | 8.324931 | 0.909756 | 8.201307 | 0.91683 |
| novel_pir979 | 0.01 | 11.85333 | 17.63667 | 10.21108 | 0.966463 | 10.78436 | 0.978147 |
| novel_pir61 | 1.273333 | 0.01 | 0.01 | -6.99247 | 0.83435 | -6.99247 | 0.845401 |
| novel_pir647 | 0.01 | 3.726667 | 5.906667 | 8.541742 | 0.91687 | 9.2062 | 0.95075 |
| novel_pir1328 | 0.01 | 0.943333 | 3.436667 | 6.559696 | 0.801016 | 8.424866 | 0.925799 |

| | | | | | | | |
|---|---|---|---|---|---|---|---|
| novel_pir980 | 0.01 | 14.12 | 17.43333 | 10.46352 | 0.969309 | 10.76763 | 0.978147 |
| novel_pir1381 | 2.343333 | 0.01 | 0.01 | -7.87242 | 0.89065 | -7.87242 | 0.900685 |
| novel_pir1600 | 0.01 | 5.903333 | 8.6 | 9.205386 | 0.942886 | 9.748193 | 0.962166 |
| novel_pir856 | 0.01 | 38.33667 | 24.24333 | 11.90451 | 0.988415 | 11.24337 | 0.982877 |
| novel_pir1001 | 0.01 | 1.183333 | 2.25 | 6.886713 | 0.827846 | 7.813781 | 0.898728 |
| novel_pir538 | 0.01 | 2.44 | 4.616667 | 7.930737 | 0.89248 | 8.850708 | 0.941455 |
| novel_pir1021 | 0.01 | 5.96 | 10.04333 | 9.219169 | 0.943293 | 9.972022 | 0.96559 |
| novel_pir504 | 0.15 | 30.69667 | 53.26333 | 7.676976 | 0.962195 | 8.472036 | 0.984181 |
| novel_pir1137 | 16.28333 | 0.01 | 0.203333 | -10.6692 | 0.971951 | -6.32341 | 0.905251 |
| novel_pir956 | 0.01 | 1.966667 | 2.766667 | 7.619609 | 0.879472 | 8.112005 | 0.912264 |
| novel_pir133 | 0.01 | 1.136667 | NA | 6.828665 | 0.825203 | NA | NA |
| novel_pir674 | 3.386667 | 0.01 | NA | -8.40372 | 0.913618 | NA | NA |
| novel_pir978 | 0.01 | 12.64333 | 17.88 | 10.30416 | 0.967276 | 10.80413 | 0.978147 |
| novel_pir1312 | 0.01 | 1.72 | 2.17 | 7.426265 | 0.864431 | 7.761551 | 0.895629 |
| novel_pir967 | 0.343333 | 50.04333 | 83.84 | 7.187424 | 0.956911 | 7.931885 | 0.982224 |
| novel_pir542 | 0.01 | 2.106667 | 4.486667 | 7.718818 | 0.883537 | 8.8095 | 0.939335 |
| novel_pir655 | 0.01 | 1.053333 | 3.543333 | 6.718818 | 0.81565 | 8.468963 | 0.926451 |
| novel_pir1000 | 0.01 | 3.103333 | 2.933333 | 8.277675 | 0.908333 | 8.196397 | 0.916667 |
| novel_pir1238 | 1.26 | 0.01 | 0.01 | -6.97728 | 0.833537 | -6.97728 | 0.844097 |
| novel_pir1100 | 0.01 | 0.94 | NA | 6.554589 | 0.801016 | NA | NA |
| novel_pir1115 | 0.01 | 5.093333 | 9.103333 | 8.992466 | 0.935569 | 9.830251 | 0.963307 |
| novel_pir529 | 0.25 | 52.12 | 87.64333 | 7.703765 | 0.969106 | 8.453572 | 0.987932 |
| novel_pir1650 | 1.443333 | 0.01 | 0.01 | -7.17326 | 0.847764 | -7.17326 | 0.858284 |
| novel_pir855 | 0.01 | 11.09333 | 10.66333 | 10.11548 | 0.964837 | 10.05844 | 0.967058 |
| novel_pir904 | 5.14 | 0.01 | NA | -9.00562 | 0.936585 | NA | NA |
| novel_pir1713 | 0.01 | 1.146667 | 3.136667 | 6.841302 | 0.825813 | 8.293088 | 0.920254 |
| novel_pir110 | 1.743333 | 0.01 | 0.01 | -7.4457 | 0.865447 | -7.4457 | 0.877365 |
| novel_pir941 | 0.01 | 1.233333 | 1.696667 | 6.946419 | 0.831707 | 7.406559 | 0.875082 |
| novel_pir507 | 0.01 | 16.47333 | 22.97 | 10.68592 | 0.972358 | 11.16554 | 0.982061 |
| novel_pir248 | 0.01 | 15.50333 | 31.36667 | 10.59836 | 0.971545 | 11.61502 | 0.987443 |
| novel_pir1121 | 0.01 | 2.726667 | 2.966667 | 8.090995 | 0.900407 | 8.212699 | 0.917971 |
| novel_pir168 | 0.01 | 2.316667 | 4.626667 | 7.855907 | 0.889837 | 8.853829 | 0.941781 |
| novel_pir1590 | 1.443333 | 0.01 | 0.01 | -7.17326 | 0.847764 | -7.17326 | 0.858284 |
| novel_pir463 | 0.01 | 1.683333 | 5.766667 | 7.395177 | 0.862398 | 9.171594 | 0.950098 |
| novel_pir523 | 0.01 | 1.61 | 6.81 | 7.330917 | 0.859959 | 9.411511 | 0.954827 |
| novel_pir573 | 0.01 | 1.556667 | 5.703333 | 7.282316 | 0.855285 | 9.155662 | 0.949772 |
| novel_pir977 | 0.466667 | 37.62333 | 57.86667 | 6.333091 | 0.914024 | 6.954196 | 0.958252 |
| novel_pir46 | 0.96 | 0.01 | 0.01 | -6.58496 | 0.804472 | -6.58496 | 0.808545 |
| novel_pir669 | 2.343333 | 0.01 | NA | -7.87242 | 0.89065 | NA | NA |
| novel_pir587 | 0.01 | 7.393333 | 23.23333 | 9.530081 | 0.951016 | 11.18198 | 0.982061 |
| novel_pir1114 | 0.01 | 2.866667 | 10.07333 | 8.16323 | 0.903659 | 9.976325 | 0.965753 |
| novel_pir1462 | 0.01 | 2.933333 | 1.373333 | 8.196397 | 0.904472 | 7.101538 | 0.854044 |

| | | | | | | | |
|---|---|---|---|---|---|---|---|
| novel_pir1501 | 0.01 | 2.203333 | NA | 7.783544 | 0.885569 | NA | NA |
| novel_pir1011 | 0.01 | 1.796667 | 1.476667 | 7.489179 | 0.869715 | 7.2062 | 0.860894 |
| hsa_piR_004150 | 19.68333 | NA | 1084.94 | NA | NA | 5.784497 | 0.866112 |
| hsa_piR_020490 | 0.183333 | NA | 13.82667 | NA | NA | 6.23684 | 0.896608 |
| hsa_piR_020548 | 0.57 | NA | 29.09667 | NA | NA | 5.673748 | 0.838715 |
| novel_pir1010 | 0.01 | NA | 1.35 | NA | NA | 7.076816 | 0.85225 |
| novel_pir1012 | 0.01 | NA | 1.746667 | NA | NA | 7.448461 | 0.878343 |
| novel_pir1013 | 0.01 | NA | 0.986667 | NA | NA | 6.624491 | 0.813112 |
| novel_pir1018 | 0.01 | NA | 1.02 | NA | NA | 6.672425 | 0.815721 |
| novel_pir1019 | 0.01 | NA | 1.18 | NA | NA | 6.882643 | 0.835943 |
| novel_pir1026 | 0.01 | NA | 1.276667 | NA | NA | 6.996238 | 0.846543 |
| novel_pir109 | 3.083333 | NA | 0.01 | NA | NA | -8.26835 | 0.919602 |
| novel_pir1119 | 0.01 | NA | 0.906667 | NA | NA | 6.5025 | 0.800228 |
| novel_pir1120 | 0.01 | NA | 1.086667 | NA | NA | 6.763766 | 0.825016 |
| novel_pir1125 | 0.01 | NA | 1.313333 | NA | NA | 7.037089 | 0.847521 |
| novel_pir1130 | 0.01 | NA | 1.38 | NA | NA | 7.108524 | 0.854371 |
| novel_pir1149 | 0.01 | NA | 0.926667 | NA | NA | 6.533979 | 0.802022 |
| novel_pir1201 | 0.01 | NA | 1.133333 | NA | NA | 6.824428 | 0.831213 |
| novel_pir1207 | 0.01 | NA | 1.193333 | NA | NA | 6.898853 | 0.836432 |
| novel_pir1244 | 0.01 | NA | 1.253333 | NA | NA | 6.969626 | 0.843607 |
| novel_pir1249 | 0.01 | NA | 1.893333 | NA | NA | 7.564785 | 0.884214 |
| novel_pir1257 | 0.01 | NA | 1.88 | NA | NA | 7.554589 | 0.883725 |
| novel_pir1267 | 0.01 | NA | 1.366667 | NA | NA | 7.094518 | 0.852903 |
| novel_pir1302 | 0.01 | NA | 2.14 | NA | NA | 7.741467 | 0.894814 |
| novel_pir1334 | 0.01 | NA | 1.596667 | NA | NA | 7.318919 | 0.868395 |
| novel_pir1338 | 0.01 | NA | 1.966667 | NA | NA | 7.619609 | 0.888943 |
| novel_pir1394 | 0.01 | NA | 1.59 | NA | NA | 7.312883 | 0.867254 |
| novel_pir1395 | 0.01 | NA | 1.726667 | NA | NA | 7.431846 | 0.877038 |
| novel_pir1398 | 0.01 | NA | 0.906667 | NA | NA | 6.5025 | 0.800228 |
| novel_pir1413 | 0.01 | NA | 1.093333 | NA | NA | 6.77259 | 0.825016 |
| novel_pir1415 | 0.01 | NA | 1.106667 | NA | NA | 6.790077 | 0.827462 |
| novel_pir1418 | 0.01 | NA | 1.83 | NA | NA | 7.5157 | 0.880952 |
| novel_pir1419 | 0.01 | NA | 2.123333 | NA | NA | 7.730187 | 0.894325 |
| novel_pir1429 | 0.01 | NA | 1.9 | NA | NA | 7.569856 | 0.885356 |
| novel_pir1437 | 0.01 | NA | 1.16 | NA | NA | 6.857981 | 0.833496 |
| novel_pir145 | 2.87 | NA | 0.01 | NA | NA | -8.16491 | 0.915851 |
| novel_pir1504 | 0.01 | NA | 1.193333 | NA | NA | 6.898853 | 0.836432 |
| novel_pir158 | 2.953333 | NA | 0.01 | NA | NA | -8.2062 | 0.917156 |
| novel_pir1635 | 0.01 | NA | 1.216667 | NA | NA | 6.92679 | 0.839693 |
| novel_pir167 | 0.01 | NA | 1.573333 | NA | NA | 7.297681 | 0.866601 |
| novel_pir1677 | 0.01 | NA | 2.056667 | NA | NA | 7.684164 | 0.89302 |
| novel_pir1694 | 0.01 | NA | 2.293333 | NA | NA | 7.841302 | 0.899543 |

| | | | | | | | |
|---|---|---|---|---|---|---|---|
| novel_pir1695 | 0.01 | NA | 1.123333 | NA | NA | 6.811642 | 0.828115 |
| novel_pir1698 | 0.01 | NA | 1.503333 | NA | NA | 7.232021 | 0.862035 |
| novel_pir1707 | 0.01 | NA | 0.946667 | NA | NA | 6.564785 | 0.80773 |
| novel_pir1715 | 0.01 | NA | 1.006667 | NA | NA | 6.653442 | 0.814416 |
| novel_pir223 | 0.01 | NA | 1.446667 | NA | NA | 7.176589 | 0.858937 |
| novel_pir281 | 0.01 | NA | 1.503333 | NA | NA | 7.232021 | 0.862035 |
| novel_pir296 | 0.01 | NA | 1.77 | NA | NA | 7.467606 | 0.879159 |
| novel_pir324 | 0.01 | NA | 1.533333 | NA | NA | 7.260528 | 0.863177 |
| novel_pir331 | 0.01 | NA | 1.53 | NA | NA | 7.257388 | 0.863177 |
| novel_pir386 | 0.01 | NA | 1.933333 | NA | NA | 7.594947 | 0.887476 |
| novel_pir399 | 0.01 | NA | 1.51 | NA | NA | 7.238405 | 0.862198 |
| novel_pir415 | 0.01 | NA | 1.536667 | NA | NA | 7.26366 | 0.86334 |
| novel_pir435 | 0.01 | NA | 1.11 | NA | NA | 6.794416 | 0.827462 |
| novel_pir436 | 0.01 | NA | 1.016667 | NA | NA | 6.667703 | 0.815721 |
| novel_pir443 | 0.01 | NA | 1.023333 | NA | NA | 6.677132 | 0.815721 |
| novel_pir450 | 0.01 | NA | 1.683333 | NA | NA | 7.395177 | 0.874755 |
| novel_pir464 | 0.01 | NA | 1.1 | NA | NA | 6.78136 | 0.826158 |
| novel_pir465 | 0.01 | NA | 1.033333 | NA | NA | 6.691162 | 0.818004 |
| novel_pir471 | 0.01 | NA | 1.19 | NA | NA | 6.894818 | 0.836432 |
| novel_pir472 | 0.683333 | NA | 43.36 | NA | NA | 5.987631 | 0.882909 |
| novel_pir479 | 0.01 | NA | 1.03 | NA | NA | 6.686501 | 0.818004 |
| novel_pir505 | 0.01 | NA | 1.006667 | NA | NA | 6.653442 | 0.814416 |
| novel_pir506 | 0.01 | NA | 1.15 | NA | NA | 6.84549 | 0.832518 |
| novel_pir508 | 0.01 | NA | 2.946667 | NA | NA | 8.20294 | 0.917156 |
| novel_pir51 | 0.01 | NA | 1.15 | NA | NA | 6.84549 | 0.832518 |
| novel_pir511 | 0.01 | NA | 1.306667 | NA | NA | 7.029747 | 0.847521 |
| novel_pir515 | 0.01 | NA | 1.593333 | NA | NA | 7.315904 | 0.867254 |
| novel_pir516 | 0.01 | NA | 2 | NA | NA | 7.643856 | 0.890411 |
| novel_pir522 | 0.01 | NA | 1.746667 | NA | NA | 7.448461 | 0.878343 |
| novel_pir533 | 0.01 | NA | 2.056667 | NA | NA | 7.684164 | 0.89302 |
| novel_pir564 | 0.01 | NA | 3.183333 | NA | NA | 8.314394 | 0.920581 |
| novel_pir566 | 0.693333 | NA | 35.49333 | NA | NA | 5.677855 | 0.84165 |
| novel_pir571 | 0.01 | NA | 1.073333 | NA | NA | 6.745954 | 0.82257 |
| novel_pir581 | 0.01 | NA | 1.106667 | NA | NA | 6.790077 | 0.827462 |
| novel_pir583 | 0.01 | NA | 1.02 | NA | NA | 6.672425 | 0.815721 |
| novel_pir590 | 0.01 | NA | 2.013333 | NA | NA | 7.653442 | 0.891226 |
| novel_pir599 | 0.01 | NA | 1.09 | NA | NA | 6.768184 | 0.825016 |
| novel_pir656 | 0.01 | NA | 1.26 | NA | NA | 6.97728 | 0.844097 |
| novel_pir706 | 0.01 | NA | 1.15 | NA | NA | 6.84549 | 0.832518 |
| novel_pir719 | 0.01 | NA | 2.05 | NA | NA | 7.67948 | 0.892694 |
| novel_pir722 | 0.01 | NA | 0.936667 | NA | NA | 6.549464 | 0.807078 |
| novel_pir731 | 0.01 | NA | 1.076667 | NA | NA | 6.750428 | 0.824364 |

| | | | | | | | |
|---|---|---|---|---|---|---|---|
| novel_pir733 | 0.01 | NA | 1.643333 | NA | NA | 7.360481 | 0.870841 |
| novel_pir788 | 0.01 | NA | 0.986667 | NA | NA | 6.624491 | 0.813112 |
| novel_pir792 | 0.01 | NA | 1.793333 | NA | NA | 7.4865 | 0.879485 |
| novel_pir804 | 0.01 | NA | 0.986667 | NA | NA | 6.624491 | 0.813112 |
| novel_pir808 | 0.01 | NA | 1.273333 | NA | NA | 6.992466 | 0.845401 |
| novel_pir822 | 0.01 | NA | 1.293333 | NA | NA | 7.01495 | 0.847032 |
| novel_pir858 | 0.01 | NA | 1.516667 | NA | NA | 7.24476 | 0.862524 |
| novel_pir924 | 0.01 | NA | 1.07 | NA | NA | 6.741467 | 0.82257 |
| novel_pir950 | 0.01 | NA | 1.023333 | NA | NA | 6.677132 | 0.815721 |
| novel_pir951 | 0.01 | NA | 2.49 | NA | NA | 7.960002 | 0.904436 |
| novel_pir963 | 0.01 | NA | 1.936667 | NA | NA | 7.597432 | 0.887476 |
| novel_pir973 | 0.823333 | NA | 48.02 | NA | NA | 5.866015 | 0.870189 |
| novel_pir974 | 0.253333 | NA | 21.40667 | NA | NA | 6.400879 | 0.918461 |

**Supplementary Table S2.** Sequences of differentially expressed known and novel piRNA.

| piRNA id | Sequence |
|---|---|
| hsa_piR_000753 | CCACCCTGAACGCGCCCGAT |
| hsa_piR_013624 | TGGGAATGCAGCCCAAAGCGGGTGGTA |
| hsa_piR_018573 | TTGGTGTATGTGCTTGGCTGAGGAGCC |
| hsa_piR_004150 | TCCCTGGTAGTCTAGTGGTTAGGATTC |
| hsa_piR_020490 | GGGGCGAAGCTACCATCTGTGGGATT |
| hsa_piR_020548 | GGTCAGTCGGTCCTGAGAGATGGGCGAGC |
| novel_pir46 | TTTCTCGCTGGGGCCTCCA |
| novel_pir51 | ACTCAATTTCTGGTCTCC |
| novel_pir61 | AATCCGAGTCACGGCACCA |
| novel_pir109 | CTCCTGGCTGGCTCGCCA |
| novel_pir110 | TCCTCCTGGCTGGCTCGCCA |
| novel_pir111 | CCTCCTGGCTGGCTCGCCA |
| novel_pir128 | GTGGTCTAGTGGCTAGGA |
| novel_pir624<br>3 | GGTGGTCTAGTGGCTAGG |
| novel_pir145 | CGGATCCCACTTCTGACACCA |
| novel_pir158 | AATCCCACTTCTGACACCA |
| novel_pir167 | CACTGACCCGGTGAGGCGGGGGGG |
| novel_pir168 | ACTGACCCGGTGAGGCGGGGGGG |
| novel_pir187 | GAATCCCACTTCTGACACCA |
| novel_pir223 | CTACGGCCATACCACCCTG |
| novel_pir248 | TTGGGTTTTAAGCAGGAGG |
| novel_pir256 | AGTGGTTAAGGCAATGGA |
| novel_pir281 | AATGCTGCTGGAGTAATTGG |
| novel_pir296 | AAAACTCCCGTGCTGATC |
| novel_pir324 | GCCTAAGGAGGGGTGAACCGGCCC |
| novel_pir329 | GCCTAAGGAGGGGTGAACCGGCC |
| novel_pir331 | CCTAAGGAGGGGTGAACCGGCC |
| novel_pir386 | CACTAAGTTCGGCATCAATATGGTGACCTCC |
| novel_pir399 | CACTAAGTTCGGCATCAATATGGTG |
| novel_pir415 | CCGATCGGGTGTCCGCACTAAGTTCGGCATCA |
| novel_pir435 | ATCGGGTGTCCGCACTAAGTTCGGC |
| novel_pir436 | CCGATCGGGTGTCCGCACTAAGTTCGG |
| novel_pir443 | GGTGTCCGCACTAAGTTCGG |
| novel_pir449 | TGCCGATCGGGTGTCCGCACTAAGTTC |
| novel_pir450 | TGCCGATCGGGTGTCCGCACTAAGTT |
| novel_pir457 | TGCCGATCGGGTGTCCGCACTA |
| novel_pir459 | TGCCGATCGGGTGTCCGCACT |
| novel_pir463 | TGCCGATCGGGTGTCCGCAC |
| novel_pir464 | TGCCGATCGGGTGTCCGCA |
| novel_pir465 | CGCTATGCCGATCGGGTGTCCGCA |
| novel_pir471 | TGCGCTATGCCGATCGGGTGTCCG |
| novel_pir472 | CGCTATGCCGATCGGGTGTCCG |
| novel_pir479 | CGCTATGCCGATCGGGTGTC |

| | |
|---|---|
| novel_pir504 | AGTTCTGGGCTGTAGTGCGCTATG |
| novel_pir505 | CTGGGCTGTAGTGCGCTATG |
| novel_pir506 | GTTCTGGGCTGTAGTGCGCTATG |
| novel_pir507 | GAGTTCTGGGCTGTAGTGCGCTATG |
| novel_pir508 | TCTGGGCTGTAGTGCGCTATG |
| novel_pir511 | TCTGGGCTGTAGTGCGCTAT |
| novel_pir512 | AGTTCTGGGCTGTAGTGCGCTAT |
| novel_pir515 | GTTCTGGGCTGTAGTGCGCTAT |
| novel_pir516 | TTCTGGGCTGTAGTGCGCTAT |
| novel_pir518 | AGTTCTGGGCTGTAGTGCGCTA |
| novel_pir519 | GGAGTTCTGGGCTGTAGTGCGCTA |
| novel_pir522 | AGGAGTTCTGGGCTGTAGTGCGCTA |
| novel_pir523 | GTTCTGGGCTGTAGTGCGCTA |
| novel_pir525 | GAGTTCTGGGCTGTAGTGCGCTA |
| novel_pir527 | GTTCTGGGCTGTAGTGCGCT |
| novel_pir528 | AGTTCTGGGCTGTAGTGCGCT |
| novel_pir529 | GAGTTCTGGGCTGTAGTGCGCT |
| novel_pir533 | AGTTCTGGGCTGTAGTGCGC |
| novel_pir535 | GAGTTCTGGGCTGTAGTGCGC |
| novel_pir536 | AGTTCTGGGCTGTAGTGCG |
| novel_pir538 | GAGTTCTGGGCTGTAGTGCG |
| novel_pir542 | AGGAGTTCTGGGCTGTAGTG |
| novel_pir564 | GTGGGAGGATCGCTTGAGCCCAGG |
| novel_pir566 | AGGTGGGAGGATCGCTTGAGCCCAGG |
| novel_pir571 | GGTGGGAGGATCGCTTGAGCCCAGG |
| novel_pir573 | AGGTGGGAGGATCGCTTGAGCCCAG |
| novel_pir580 | AGGTGGGAGGATCGCTTGAGCCCA |
| novel_pir581 | GTGGGAGGATCGCTTGAGCCCA |
| novel_pir583 | TGGGAGGATCGCTTGAGCCCA |
| novel_pir587 | AGGTGGGAGGATCGCTTGAGCCC |
| novel_pir590 | AGGTGGGAGGATCGCTTGAGCC |
| novel_pir599 | GGCTGAGGTGGGAGGATCGCTTG |
| novel_pir607 | GGCTGAGGTGGGAGGATCG |
| novel_pir647 | GGTTTTAAGCAGGAGGTG |
| novel_pir655 | ATTGGAGGGCAAGTCTGGTG |
| novel_pir656 | TTGGAGGGCAAGTCTGGTG |
| novel_pir669 | CTGCTTCACTTGACTAGCCTTA |
| novel_pir674 | TGCTTCACTTGACTAGCCTTA |
| novel_pir706 | CTTGATCCTGAAGCAGCT |
| novel_pir719 | CCATACCACCCTGAACGCG |
| novel_pir722 | CCATACCACCCTGAACGCGCCCGATC |
| novel_pir731 | CATACCACCCTGAACGCGCCCGAT |
| novel_pir733 | CATACCACCCTGAACGCGCCCGATC |
| novel_pir772 | TCCCGGGTTTCGGCACCA |
| novel_pir788 | CAGGGGTAGAGCACTGGT |
| novel_pir792 | AAGGTGAGATTTTGGGCA |

| | |
|---|---|
| novel_pir797 | AGTCAGAGGAGAAGGAGG |
| novel_pir804 | CTTGATCACTGTGAAAAAT |
| novel_pir808 | TCTTGGTTTTTGTCTTCTT |
| novel_pir822 | TATCTTGCTTTATTTTCTTCA |
| novel_pir855 | GTTTGTGTGTTGTTGGCAG |
| novel_pir856 | TTTGTGTGTTGTTGGCAG |
| novel_pir857 | GTTTGTGTGTTGTTGGCA |
| novel_pir858 | CAGAAGACTTATTGCTGTTGT |
| novel_pir904 | TTCTCCTGGCTGGCTCGCCA |
| novel_pir905 | TCTCCTGGCTGGCTCGCCA |
| novel_pir924 | TGATTTCTGCCCAGTGCT |
| novel_pir935 | AGACATTTGGTGTATGTGCTTGGCTG |
| novel_pir941 | GACATTTGGTGTATGTGCTTGGCT |
| novel_pir950 | CATTTGGTGTATGTGCTTG |
| novel_pir951 | CATTTGGTGTATGTGCTTGGCT |
| novel_pir952 | CATTTGGTGTATGTGCTTGGC |
| novel_pir955 | ATTTGGTGTATGTGCTTGGCTG |
| novel_pir956 | ATTTGGTGTATGTGCTTGGCTGAGGA |
| novel_pir957 | ATTTGGTGTATGTGCTTGGCTGAGGAG |
| novel_pir960 | ATTTGGTGTATGTGCTTG |
| novel_pir963 | ATTTGGTGTATGTGCTTGGCTGA |
| novel_pir965 | TTTGGTGTATGTGCTTGGCTGAG |
| novel_pir967 | TTTGGTGTATGTGCTTGGCTG |
| novel_pir969 | TTTGGTGTATGTGCTTGG |
| novel_pir970 | TTTGGTGTATGTGCTTGGCTGAGGAG |
| novel_pir971 | TTTGGTGTATGTGCTTGGCTGAGGAGCCA |
| novel_pir972 | TTTGGTGTATGTGCTTGGCTGA |
| novel_pir973 | TTTGGTGTATGTGCTTGGC |
| novel_pir974 | TTTGGTGTATGTGCTTGGCT |
| novel_pir975 | TTTGGTGTATGTGCTTGGCTGAGGA |
| novel_pir976 | TTGGTGTATGTGCTTGGCT |
| novel_pir977 | TTGGTGTATGTGCTTGGC |
| novel_pir978 | TTGGTGTATGTGCTTGGCTGAGG |
| novel_pir979 | TTGGTGTATGTGCTTGGCTGAGGA |
| novel_pir980 | TTGGTGTATGTGCTTGGCTGAGGAG |
| novel_pir981 | TTGGTGTATGTGCTTGGCTGA |
| novel_pir982 | TTGGTGTATGTGCTTGGCTGAGGAGCCAA |
| novel_pir983 | TTGGTGTATGTGCTTGGCTGAG |
| novel_pir984 | TTGGTGTATGTGCTTGGCTG |
| novel_pir996 | GGTGTATGTGCTTGGCTG |
| novel_pir998 | GTGTATGTGCTTGGCTGAGGAGCCA |
| novel_pir999 | GTGTATGTGCTTGGCTGAGGAG |
| novel_pir1000 | GTGTATGTGCTTGGCTGAGG |
| novel_pir1001 | GTGTATGTGCTTGGCTGAGGAGCC |
| novel_pir1002 | GTGTATGTGCTTGGCTGAG |
| novel_pir1003 | GTGTATGTGCTTGGCTGA |

| | |
|---|---|
| novel_pir1004 | GTGTATGTGCTTGGCTGAGGA |
| novel_pir1010 | TGTATGTGCTTGGCTGAGGAG |
| novel_pir1011 | TGTATGTGCTTGGCTGAGGA |
| novel_pir1012 | TGTATGTGCTTGGCTGAGGAGCC |
| novel_pir1013 | GTATGTGCTTGGCTGAGGAGCC |
| novel_pir1018 | GTATGTGCTTGGCTGAGGAGCCA |
| novel_pir1019 | TATGTGCTTGGCTGAGGAGC |
| novel_pir1021 | TATGTGCTTGGCTGAGGAG |
| novel_pir1023 | TATGTGCTTGGCTGAGGA |
| novel_pir1026 | TATGTGCTTGGCTGAGGAGCCAA |
| novel_pir1027 | TATGTGCTTGGCTGAGGAGCC |
| novel_pir1028 | TATGTGCTTGGCTGAGGAGCCA |
| novel_pir1100 | AATGTGGCGTACGGAAGAC |
| novel_pir1102 | ATGTGGCGTACGGAAGAC |
| novel_pir1103 | ATGTGGCGTACGGAAGACC |
| novel_pir1114 | CCCAAGTCCTTCTGATCGAG |
| novel_pir1115 | CCCAAGTCCTTCTGATCGAGG |
| novel_pir1117 | CCAAGTCCTTCTGATCGAGG |
| novel_pir1118 | CAAGTCCTTCTGATCGAGG |
| novel_pir1119 | CAAGTCCTTCTGATCGAG |
| novel_pir1120 | AAGTCCTTCTGATCGAGG |
| novel_pir1121 | GGAAATGTGGCGTACGGAA |
| novel_pir1122 | GGAAATGTGGCGTACGGA |
| novel_pir1125 | AAAAAAAGAAGTCAGAGG |
| novel_pir1130 | CTAGGTGGTAGATATGGT |
| novel_pir1136 | TTCCCCGTACTGGCCACCA |
| novel_pir1137 | GTTCCCCGTACTGGCCACCA |
| novel_pir1149 | AGCAGGGTCGGGCCTGGTT |
| novel_pir1201 | TCAGTAGTTAGTTTATTTACA |
| novel_pir1207 | TTTGGAAAAGTAGCTTTGG |
| novel_pir1229 | TTGGGTTTTAAGCAGGAGGTG |
| novel_pir1231 | TTGGGTTTTAAGCAGGAGGT |
| novel_pir1233 | TGGGTTTTAAGCAGGAGGTG |
| novel_pir1238 | ATCCCTTCGTGGTTGCCA |
| novel_pir1244 | AATGGTTGGAAAAGTTCA |
| novel_pir1246 | CATCCCACTTCTGACACCA |
| novel_pir1249 | TGTGTCCTCTGCTGTTTCT |
| novel_pir1257 | GCCTGGATAGCTCAGTCGGTAGAGCA |
| novel_pir1259 | GCCTGGATAGCTCAGTCGGTAGAGC |
| novel_pir1264 | GATAGCTCAGTCGGTAGAG |
| novel_pir1267 | ATAGCTCAGTCGGTAGAG |
| novel_pir1302 | TGTAATGGTTAGCACTCTGG |
| novel_pir1312 | ATGGTTAGCACTCTGGACTCT |
| novel_pir1315 | ATGGTTAGCACTCTGGACTCTG |
| novel_pir1324 | TTTGTAGTTGATTGAATCT |
| novel_pir1328 | TACTTGTTGGATTGTTGA |

| | |
|---|---|
| novel_pir1334 | TCTGTTATTGATCTTGGCA |
| novel_pir1338 | TCAGGTTCAGGAGCAGGT |
| novel_pir1381 | GTATCCCACTTCTGACACCA |
| novel_pir1391 | AACCAAGAGAGTAGCACT |
| novel_pir1394 | TCTTTGTTCTTGTGTTGCT |
| novel_pir1395 | TGGTGTTAGAATGTTCATT |
| novel_pir1398 | GTGGTATAGTGGTGAGCATA |
| novel_pir1411 | CTCTCTCTCTCTCTCCCCCGCT |
| novel_pir1413 | GATGGGGAGTTTGGCTGGGGC |
| novel_pir1415 | TGGGGAGTTTGGCTGGGG |
| novel_pir1418 | TAATCTTTTGCTGCTCTTTT |
| novel_pir1419 | TAATCTTTTGCTGCTCTTT |
| novel_pir1429 | ACTTGGTCTCTTGTTTGT |
| novel_pir1437 | TTCATCTTGGATTATCTGG |
| novel_pir1462 | AAGGGAACGGACTTGAAG |
| novel_pir1501 | GTGGTCTAGTGGTTAGGA |
| novel_pir1504 | TCTTTTGTGGTTGTTGTTG |
| novel_pir1575 | ACTGAAGATCTAAAGGTCCCTG |
| novel_pir1590 | GATCCCACTCCTGACACCA |
| novel_pir1600 | GATTCCGTGGGTGGTGGTG |
| novel_pir1601 | ATTCCGTGGGTGGTGGTG |
| novel_pir1618 | TATCTCGCTGGGGCCTCCA |
| novel_pir1627 | TTACAAAGGAACAGTTGGC |
| novel_pir1628 | TTACAAAGGAACAGTTGG |
| novel_pir1635 | CCTATTCAGTATGGTTGT |
| novel_pir1650 | CAATCCCGGACGAGCCCCCA |
| novel_pir1677 | ATGTTGTTCTACTTTTTGT |
| novel_pir1685 | GTACGTAGCAGAGCAGCTCCCTCGCTG |
| novel_pir1686 | GTACGTAGCAGAGCAGCTCCCTCGCT |
| novel_pir1694 | ACGTAGCAGAGCAGCTCCCTCGCTG |
| novel_pir1695 | CGTAGCAGAGCAGCTCCCTCGCTG |
| novel_pir1698 | CGTAGCAGAGCAGCTCCCTCGCT |
| novel_pir1703 | ATCTATTGAAAGTCAGCCCT |
| novel_pir1707 | TCTATTGAAAGTCAGCCCT |
| novel_pir1713 | ATGATGTGTTGTTGCCAT |
| novel_pir1715 | CATGCCTCAGAATCACTG |
